# Supplementary material for: The Lions Sports Academy TackleTEK Tool: The intra-and inter-coach reliability of assessing tackling competency in rugby union
Source: PLoS One. 2026 Apr 24;21(4):e0346881. doi: 10.1371/journal.pone.0346881 (PMC13108810; doi:10.1371/journal.pone.0346881)
Supplement: Attachment — Submitted filename: REVIEWERS REPLY_R2.pdf [file pone.0346881.s003.pdf]

PEER REVIEW ROUND 2

The Lions Sports Academy TackleTEK Tool: The intra-and inter-coach reliability of assessing tackling competency in rugby union

---

INTRODUCTION:

Dear authors,

In the following review, I have critically appraised the responses provided to the first review. Considering the extensive work you have done and the significant improvements this carries with it, I have to sincerely congratulate you on your diligence and the dedication to improve your research. These efforts have resulted in a manuscript of higher quality. In addition to that, I believe it would also be more productive to refrain from using any emotionally loaded language and ad hominem fallacies within your replies.

Notwithstanding the improvements that were made and the clarifications that were provided, there are still some concerns that need to be addressed. Some of them are minor details, other concerns require confirmation or further clarification, and some require more impactful changes. Overall, please consider that this manuscript is closer to publication and simultaneously, the critical nature of this peer review is intended as such. This journal has high standards and good intentions do not automatically lead to immediate successful outcomes. Which does not take away from the authors' intent.

Please, also recognise that some methodology used in prior published research, used as reference within this manuscript, does not inherently form an adequate basis of support, if the methodology was inadequate or suboptimal to begin with. Consequently, the current research is being reviewed on its own merits, and critically assessed, irrespective of former publications perhaps being less optimal. The aim is to accentuate this manuscript's strengths while mitigate its limitation and upkeep the scientific rigour for which PLOS ONE is known.

Considering the extent of the reviewed and improved manuscript, in the first section below (p. 2), the authors can find an integral text addressing most pressing issues, as chronologically presented in the former document and addressed by the authors (round 1). In-depth replies to the general comments are provided and may also overlap with and address specific subsequent sections within the manuscript. Responses that were not addressed were found less essential or sufficiently buttressed by the authors at this current stage. Following this in-depth appraisal, the authors can find a section with minor amendments to specific phrases as well.

Overall, well done.

Kind regards.

2nd APPRAISAL GENERAL IN-DEPTH COMMENTS:

RESPONSE TO THE AUTHORS' REPLIES IN THE FIRST REVIEW

To address the first part of your response; I do not see the purpose of this comment, as it is incorrect and the author seems to know this; on several occasions in this review the author contradicts this statement of there not being any known validated instruments or processes by stating "an established framework has been followed…", "the recommended process…", "current process follows the recommended one", and others alike. In addition, some evidence (references) is provided for it, such as what follows below. Your stance on this matter is therefore inconsistent.

To my knowledge and for your information, the Bangor Rugby Assessment tool is currently the most practically useful and strongest scientifically validated tool with good reliability (doi: 10.3389/fspor.2025.1568302). As this instrument was specifically developed for technical and tactical aptitude in rugby union development pathways, I recommend integrating this into your manuscript.

Furthermore, as you put forward, den Hollander et al. (2019) provide some backing (with moderate evidence of construct validity) for the current study. However, Gabbett (2008) is questionable as a reliability study, indeed. Moreover, that study holds limited statistical power and carries little weight in terms of evidence of intra- or interrater reliability. Their main aim was not to prove any form of reliability, rather, to investigate the influence of fatigue on tackling technique and relate it to physiological capacity variables.

In addition to these studies, the *Tackle Ready* framework could provide guidance in developing and validating an instrument, such as the one under review. Further to that, parallel research, such as in football (doi: 10.3389/fpsyg.2019.00022) or tennis (doi: 10.3389/fpsyg.2018.02418) can be very useful as a source of methodological guidance.

Towards the process for validating skill-related competency tools; this process is not fully rigid, but nevertheless fairly well-established, indeed:

1. **Conceptualise the initial tool design**: thorough literature review (ID key technical or tactical variables,…), expert group feedback (create content validity through consolidating observational criterion, working definitions, etc); drafting tool
2. **Validity testing:** (face validity), construct validity, criterion validity (compare to 'gold standard' or alternatively, objective standard such as expert observation of successful/safe match tackles, e.g.)
3. **Reliability testing**: inter- and intra-rater. As per the aim of the current investigation.
4. **Refinements**: ecological validity, practical/logistical utility,… and necessary modifications.

Please look further into the necessary details.

I believe that, in the current study and the pilot study, various of these components were targeted. But perhaps not all components were present, designed, or executed to the utmost extent, in my view. If, however, all components were diligently executed, then the communication and transparency regarding its development is lacking; other than a narrative general description in the current study, I have not seen compelling evidence of the developmental workflow of this instrument, specifically, and its (partial) validation (i.e., nr. 1 and especially nr. 2 in the process described above).

Indeed, the current study aim is to test the instrument's reliability. I realise that. But considering its reliability (are the measurements consistent?) is part of the greater process of the tool's testing and evaluation, which is normally preceded by its validity testing (does it measure what it is supposed to measure?), it is perfectly reasonable and logical to have an insight into this component. Otherwise, without evidence of the instrument measuring what it is supposed to measure, its reliability is rendered mute.

Thank you for adding the reference (31) of the ECSS 2024 Book of Abstracts. This is interesting information in itself. However, its content does not address the above issue. This information does not seem to be available. Can the authors please include or disclose their systematic approach to developing the TackleTEK instrument, other than generically referencing "pilot data". Preferably, a step-by-step workflow document addressing the design phase and validation subcomponents, as outlined above, would be feasible. This can be done within the manuscript or as an additional document made available alongside this manuscript.

With that in mind, it is true that the process for developing such an instrument optimally is not a simple undertaking, nor is it fully uniform across the literature. Many studies do not disclose their systematic developmental approach. Rather, oftentimes, "experts" and/or practitioners are *mentioned* to have developed the technical or tactical criteria, e.g., Gabbett (2008), which lacks transparency and thus, possibly, methodological rigidity. Alternatively, technical criteria may be blindly copied or adapted from prior research which is then referenced. Simply because it was published; e.g., den Hollander et al. (2019) citing Hendricks et al. (2015), Burger et al. (2016), and Davidow et al. (2018).

Please, bear in mind that such research may or may not be adequate, in spite of its publication! Simply referencing a published study without exactly framing why or which part of that evidence buttresses your case, does not inherently qualify it as an adequate base for your research. The body of literature, in some cases, is rife with suboptimal methodology. And methodological shortcomings are transmissible, indeed. Please, select your references thoroughly and thoughtfully, to have your research stand out.

These (existing) skill evaluation instruments may therefore, or may not, have sufficient validity and/or reliability. Consequently, using the same or similar (partial) methods, may or may not be optimal. To confirm this, the original validation and/or reliability study should be referenced, specifically. This original research should be understood and found to be methodologically sound. Additionally, or alternatively, the (novel or improved) developmental process (such as in the current study) should be maximally disclosed.

This form of critical appraisal, such as in the current peer review process, is in function of maximising overall transparency for the reader, such that interpretation and replication can happen to the utmost extent (Harrison et al, 2021 - https://www.arthroscopyjournal.org/article/S0749-8063(21)00821-5/fulltext; Harrison et al., 2022 - https://link.springer.com/article/10.1007/s00167-022-06893-9; Mesquida et al., 2022 - https://royalsocietypublishing.org/doi/full/10.1098/rsos.220946 and many others).

Please, understand this well; I understand and agree that the methodological approach in the current study regarding the statistical techniques employed is superior to some, and perhaps even most studies referenced by the authors. Yet, the choice of statistical methods and the execution thereof, are only part of an optimal reliability study. This reliability, in turn, is only part of the instrument's greater evaluation. Since this instrument is under examination and it was

developed using pilot data, it is reasonable in terms of transparency, to request a more precise insight into its systematic development. Could the authors please provide elaboration on the validation process up to the reliability testing as outlined above? Otherwise, it leaves room for the reader to interpret that the instrument's validity was assumed (not proven) to collect pilot data which then served as the basis for its reliability testing.

Can the authors confirm that my interpretation of the terminology "analytical goals" in the current study is accurate? As you are aware, you borrow this term form Atkinson and Nevill (1998) and you mention it generously. Atkinson and Nevill (1998) see *analytical goal*-setting as the requirement for effective practical use of the measurement tool. They do not formally define it. In turn, they sourced it from Ross & Fraser (1993) and Fraser & Peterson (1990), who developed it within a clinical context. It is wise to assume that the reader will not automatically be aware of the content or the terminology surrounding your research and the extensive body of literature supporting your research; they were not involved, and it may not be their (specific) area of research or expertise. Consequently, it is good practice to catalyse the readers' understanding of your research, by facilitating an optimal interpretation of the terminology used.

In the context of your study, I believe an early and concise clarification (in straightforward language) of your interpretation of Atkinson and Nevill's "analytical goals" (perhaps frame within the original research of Fraser & Peterson (1990) and Ross & Fraser (1993)), would be helpful to expedite the comprehension of the methods.

I fear that, in my original interpretation of the manuscript, I understood you were somehow referring to 4 of the 18-point criteria (randomly chosen) stated in Table 1 (p.7, L 158). This would beg the question of why you stated that other studies using similar technical criteria to evaluate players' skills (e.g., Gabbett (2008) did not develop "a-priori analytical goals".

My current understanding is that your "analytical goal" is referring to the improvement in composite tackling scores (to the magnitude) of four arbitrary units, when using the 18-point criteria instrument. As that is the median improvement which resulted from the application of the TackleTEK programme, in the empirical pilot data presented at ECSS 2024. Correct? If so, please consider rewriting that section in such a manner that readers are more likely to immediately understand what you are trying to communicate. There may be individual differences in comprehension between people, of course. But please bear in mind that the burden of translating esoterical concepts into a more easily digestible message, falls onto the authors. As the saying goes; expertise is making complex matters look simply.

As mentioned in the original review, the statistical methods in this manuscript were considered and they are, in and of themselves, not the core issue I put forward. In fact, I stated that they are adequate and seem well executed. Your point about these statistical methods being a valuable addition, going beyond what the body of literature had provided, has certain merit.

The "evidence-based analytical goals" stem from the (unreviewed but presented?) pilot data, on which is currently not being fully shared. The ECSS 2024 book of abstracts offer limited insight. The current study and the available data are insufficient for replication of this research. This targets not just recreation of the design in an alternative context but includes replication of the statistical processes in the current and the pilot study. The availability of this (raw, anonymised) data serves as a potential base for literature reviews and meta-analyses.

The current situation is a decent starting point but as argued, sharing the (anonymised) pilot data, either in a virtual depository, additional file to the manuscript, or making it available on request

(can be mentioned in the manuscript), at the very least, would be a critical component in terms of the necessary transparency for replication of this research, and thus greatly improve its strength.

I understand the author's view that the players are the participants according to the classic interpretation of such a tackle skill-related set up. Especially, since the original pilot study aimed at quantifying improvement in tackle skill-competency. However, this is not the pilot study. It is the assessment tool that was specifically developed, and which is being operated by the coaches, that is under investigation. The coaches can therefore not be part of the instrument. In reliability studies the participants are defined as the individuals who are providing the data (such as two or more different people who conduct the same evaluation, or one person rating the same thing at different times). The data in question in this current study, are the tackle ratings, not the tackles or players themselves. The coaches are therefore technically the participants in function of establishing the instrument's reliability. The players (or the players' tackles) can, in turn, be considered the conditions that raters are exposed to.

I understand that, in line with previous research, the players' details need to be described under the participants section. However, I would like to see the identification/description of the three raters (first sentence under "Familiarisation of raters") included within the participant's section. Further to the topic of the raters, a larger sample size of raters benefits the overall power of the investigation, as in most investigations. This is in addition to having an adequate number of tackles to be assessed by these raters, as is the case in the current study. This is irrespective of some of the cited reliability studies also having limited number of raters; *two wrongs don't make a right* – concept. Especially considering the authors state that the three raters were purposefully selected from three different tear levels. So, please add a statement outlining this limitation, specifically (i.e., along the lines of "These conclusions should be interpreted with reservations considering the limited number of raters per tear level." And "future research should…"). For the same reasons and as you have already incorporated a few lines regarding replication on a larger scale into the conclusion, I would strongly advise adapting the title of this manuscript to be more nuanced (see further down).

In my view, selecting three coaches from three 'levels' is a risky approach to the design. It puts the study results at greater risk of being affected by impactful variables, such as experience and expertise, without being able to strongly discriminate for it as there is only one rater per defining category. It is therefore very not possible to ascertain whether any discrepancies (which there clearly were, especially between tier one/two and tier three), are actually due to this defining characteristic. With multiple raters per tier group, not only would it have provided more statistical power, any trends within and between rater subcategories (tier group) would have been clearer. However, I understand that this is not practically rectifiable post-hoc.

Considering the statistical discrepancies reported between mainly the tier 3 rater on the one hand, and the tier 1 and tier 2 rater on the other, for intra- and inter-rater reliability, as well as for the intra-coach subcomponent analysis, the conclusion (in the abstract especially) needs to be amended to be more nuanced. The discussion section, which to your credit, is an outstandingly well-written, needs to be represented more adequately in the conclusion, Based on the data and the various discrepancies you correctly outline in the results and the discussion, especially, we cannot simply and solely conclude that "Overall, the levels of agreement…demonstrate that the TackleTEK Tool can be used to assess changes in tackling competency in youth rugby union.

In fact, I have to remind the authors that it was pointed out to me that a multiple tier-level rater sample was purposefully selected and part of the research question. It is therefore correct in terms of keeping the integrity of the study, to clearly state the *negative* outcome within that self-selected context. A statement in terms of: "Within the specific context of this study, the TackleTEK Tool did not demonstrate sufficient overall reliability to be applied across all coaching levels." Is essential. This can be followed up by the more nuanced sub-conclusions that are already present.

Alternatively, a statement such as "As a consequence, the tackleTEK tool was not found to be optimally reliable across all coaching levels but may be sufficiently reliable for the use of tier one and tier two coaches to assess changes in tackling competency in youth rugby union. Further research with larger samples of raters within different coaching levels is needed to strengthen these conclusions." would also be adequate. It is important that the reader clearly understands that this tool, although with its clear merits and practical utility, is not a so called one-size-fits-all. And that more research is needed to elaborate and confirm these preliminary findings. Please add this clear statement to the abstract and in the conclusion section.

As the authors state that more research on various levels is needed, and that larger data collections are currently ongoing to further strengthen the reliability of this instrument, I would strongly suggest rephrasing the title more holistically apt; e.g., "A preliminary investigation into the intra- and inter-coach reliability of measuring tackle competency with the Lions Sports Academy TackleTEK Tool by three multi-level coaches" This incorporates the limited number of raters and points out that further research will be needed, as well as immediately indicating the broad scope of a multi-level rater assessments.

In addition to the added details (L125-126): "All data collection was performed during the school term from September to April outdoors, between the hours of 2-4 pm, as part of their regular game lessons.", it is good practice to provide an overview of the exact dates on which data was collected, together with the meteorological and environmental conditions (temperature range, rainfall (mm), atmospheric pressure, natural/synthetic pitch).

With regards to the authors' statements of the existing data restrictions and the reviewer fully being able to replicate the methods; no, not quite, as certain specific details are missing (as requested above). As also addressed above, replication of the statistical processes in this current study should be achievable for other researchers, in terms of further peer-review, literature review, and meta-analyses. Ethical approval should not stand in the way of fully anonymised raw data sets. It is essential for the scientific method to recreate and replicate former studies. The case for its importance was already argued above ((Harrison et al, 2021 - https://www.arthroscopyjournal.org/article/S0749-8063(21)00821-5/fulltext; Harrison et al., 2022 - https://link.springer.com/article/10.1007/s00167-022-06893-9; Mesquida et al., 2022 - https://royalsocietypublishing.org/doi/full/10.1098/rsos.220946 and many others). Please, also consider the guidelines for het review process https://journals.plos.org/plosone/s/reviewer-guidelines#loc-writing-the-review) and the guidelines on data reporting for submission (https://journals.plos.org/plosone/s/submission-guidelines#loc-data-reporting).

To the authors' second to last reply; please, be aware that various forms of bias can be inherent to certain research situations, to our liking or not. For example, investment bias and incentive-based bias, among other forms of bias, have a real potential to interfere with the scientific process. These are logical and well-considered concerns for which I had carefully selected the

wording that was used, such as “perhaps” and “unconscious”. Biases are not always an “others” thing.

In light of the authors’ practical set up and liaison (cf. Non-random sampling, name of the instrument,...) these concerns are reasonable and should be able to be discussed openly to buttress this study and optimise this manuscript’s quality. It is therefore most unwise to reply in an ad hominem fashion with emotionally loaded language. Assuming unsubstantiated ignorance or malevolence is most unproductive. Please, refrain from convoluting this collegial process with your personal feelings. It is the authors’ research that is under examination, not the reviewer. The burden of proof is on the authors. And this critical appraisal is aimed at optimising the outcome.

We are now closer because of it.

2nd APPRAISAL MINOR CORRECTIONS:

ABSTRACT

L25

Swap “to” with “with (real-world)”

L39

Move “consistently” after “subcomponents”

INTRODUCTION

L48

Move “in research” after “playing levels”

L49

Ad comma after the references

L78

Add comma after i.e.

L86

...offers a more robust statistical method then... what/as compared to what? Make explicit.

L90

Addition: shows the range within which 95% of the differences are expected to occur...

L94

“standardised”: as addressed above, please provide evidence of structural development of the tool (i.e., workflow, validation,..). I understand the finished product “shows” a standardised

instrument. But currently, this is just a word in the manuscript with no proof of its developmental validity.

L95

Co-developed is the right spelling, I believe.

User+s

L96

Change “like” with “ in line with”

L98

Recommend + ed

L102

Consider rewording to: demonstrated the potential for a median improvement in whole (composite) tackling skill to the magnitude of four AU…

105

Add “in” before “the current study”

Change to: …a change equal to or less than four AU was determined as the”

L110-111

Consider adding “potential” before “available units”

115

Add comma after i.e.

METHODS

Design

L146

Delete references 11, 15 after “assessments”

Replace “was” with “were”

L147

Co-developed

Delete “with”

Statistical analysis

L220

Replace “like” with “in line with”

DISCUSSION

L357

Add "for this subpopulation of coaches." after "provided"

Add comma after i.e.

L364

Add comma after i.e.

CONCLUSION

L458

Only "evidence-based" if data and workflow is fully transparent. Alternatively, replace by "empirical". Ideally, please make data available.

L459

Consider changing consistent with "acceptable"

L463

Replace "likely" with "plausible", as no real (statistical) evidence of likelihood (magnitude-based inferences) available and only one coach.

L468

Add "by lower and medium level coaches" (or something in those terms) after "...can be used".

L470-471

Rephrase: "...other playing levels to investigate its reliability, with appropriate analytical goals, would increase its robustness for general use throughout rugby union."
