# Supplementary material for: The Lions Sports Academy TackleTEK Tool: The intra-and inter-coach reliability of assessing tackling competency in rugby union
Source: PLoS One. 2026 Apr 24;21(4):e0346881. doi: 10.1371/journal.pone.0346881 (PMC13108810; doi:10.1371/journal.pone.0346881)
Supplement: Attachment — Submitted filename: Peer review comments_TackleTEK.docx [file pone.0346881.s001.docx]

# Peer review

**GENERAL COMMENTS**

Dear authors, dear editor,

Thank you for the opportunity to review this manuscript. The development of youth rugby (skill) practice is an important aim within this research field, indeed. For this reason, I want to first and foremost commend the authors on their efforts to further the development of this research topic. I hope the sport can benefit from their continued contributions.

In the current study manuscript, there are various positive aspects. The overall intent to obtain a reliable measuring tool, examining both intra- and inter-rater reliability, is good practice. The statistical analysis is largely adequate and seems well-executed and reported. Conversely, the in-depth examination of the introduction, methods, and results section, have raised a number of serious concerns regarding the methodological design and development of this study. In addition, I have secondary concerns regarding the clarity with which certain sections of the manuscript are written. In conclusion, the information presented did not lead me to agree with the overall conclusion of this study.

The manuscript would benefit from greater methodological rigour. More attention should go towards precision and contextualisation of the information conveyed, such as pinpointing the particulars of the references used, as opposed to general lump-referencing or using blanket statements without precisely buttressing the argument for which the reference is integrated. The provision of more depth and appropriate nuance regarding the evidence used to frame the current research more precisely will facilitate the reader’s understanding of the current study and highlight the authors’ expertise. Lastly, greater attention should go towards correct and appropriate academic writing, regarding terminology, syntaxing, and the use of appropriate punctuation. These issues convolute the information presented. In my opinion, the manuscript should be restructured and rewritten to optimise the understanding of the actual protocol and its connection to and framing within the prior pilot study.

The main concerns regarding the methodological design and execution of this research study entail:

- The efficacy of the original pilot study (during which the tackling skill component of the TackleTEK programme was collected) was assessed using this non-validated tool, which is being investigated post hoc, in the current study, for its reliability.
- Based on this original pilot data for which this tool was used, the progress shown in four of eighteen ‘units’ was, seemingly arbitrarily, deemed adequate as the analytical goals for the current study. Apart from the arbitrary nature, I fear these first two points demonstrate a form of circular reasoning.
- Although the participants were defined as being the youth players, as this is a reliability study, the raters are arguably the subjects under investigation. The number of raters (3) is limited. In addition, these three raters differ greatly in their perceived coaching expertise, their only reported differentiating characteristic. This heterogeneity in experience level was however not accounted for as a confounder, and practically dismissed in the conclusion with a caveat. I would have liked to see a greater number of raters within each of the three tier-levels to account for this effect and strengthen this study’s generalisability.
- Using duplicates of video footage samples (25x2) to inflate the sample size (50), even though from a different angle, is a questionable practice. Especially considering the availability of sufficient original cases (206).
- There is a complete lack of detail regarding the context in which the original player data was collected during more than two and a half years. Consequently, there is no way to assess the in-practice standardisation of the protocol.
- The extensive familiarisation process undertaken with the raters by the lead researcher raises the question to what degree the outcome was influences by explicitly making the interpretation of the tool’s criteria as uniform as possible; a quality that would be inherent to a valid and reliable measuring tool. The concern is that its ability to be extrapolated to in-practice youth rugby is therefore compromised.
- The choice to use multi-angle video footage (with ad libitum rewatch) instead of live assessment of tackling skill also begs the question to which degree these outcomes are generalisable to youth rugby practice, at all levels, which to my understanding is the ultimate aim.
- Data restrictions apply and it seems anonymised data is also not available. Therefore, these already limited results are not reproducible.

Regarding the conclusion of this study, within its design, the results show select aspects of reliability, indeed. Notwithstanding, I do not agree that the information presented unambiguously shows that the tackleTEK tool is an overall reliable method for assessing changes in tackling competency. This is largely based on the questionable methodological design issues, as outlined above.

I believe this study may be a worthwhile addition to a thesis, if its contextual limitations are appropriately framed within the overarching research. However, for the reasons discussed, I am afraid I cannot recommend this manuscript for publication in PLOS ONE.

Please find further review details below.

**ABSTRACT**

Line 25-26: Strangely worded: “...however, it is important to establish the reliability of these tests to understand the capacity to identify change across time.”

This specific test or all such tests? The tool’s capacity to identify change? Change of what? Be specific, please. I believe you mean to say, you want to be able to confidently (reliably) identify change in tackling competency across time, using this specific test. Therefore, the testing tool needs to be reliable, correct? For instance, an unreliable tool would perhaps be more prone to “identify change”, by its very nature. So too could an invalid testing tool, by not measuring the variable of interest effectively. Please rephrase this sentence.

Line 29-30: “Analytical goals were developed…” This sentence is a filler with no added benefit to understanding the design, as it does not hold any useful information at this point in the manuscript; what analytical goals, why, how were they developed, and by whom? Please frame it so that its importance becomes apparent. Or alternatively leave it out if redundant.

Line 30: Subcomponent, no hyphen, is the more common spelling. Both allowed, though.

Line 33: Tacking missing an “l”.

Line 34-35: “the analytical goal of ≤4 points” at this point in the manuscript does not hold much meaning to the reader, as it has not been explained or framed. This statement assumes inside information is present before the readers gets to the body of the article. Please provide some clarification and/or rephrase.

Line 37-38: “Intra-coach sub-component analysis showed the tier one and tier two coach to repeatably score all sub-components (P > 0.05); however,…”. So, all subcomponents were scored reliably alike, or consistently reliable, yet non-significantly so? What are you trying to convey exactly? “Repeatably” is not the optimal way to infer reliability. First off, it sounds as if the act of scoring subcomponents by coaches itself was the measurement of interest, not the reliability of the scores. Additionally, provide an explanation for the p-value. Please rephrase.

**INTRODUCTION**

General note:

There is a need to define, early on, what the authors mean by the prevalently used terminology of “(sub-component) analytical goals”. What exactly do you mean by this wording within your research? Further to that, terminology such as “sub-components” and “arbitrary units” could be framed more clearly. Clarifying such terminology will greatly facilitate the readers understanding throughout, from the start. Assume that the reader does not know any of the research you aim to share.

L46-47: This is an inaccurate representation of the literature referenced, or at least lacking in nuance; skill-testing is described as a useful tool for differentiation and ID identification purposes. The literature surrounding this broad topic (in various sports, not solely rugby union) has steadily increased, indeed. Consequently, so have the protocols and frameworks provided. However, the implementation of these protocols and frameworks are not commonplace and are mainly reserved for talented youth programmes. To my knowledge, no study has investigated the implementation, i.e., the prevalence of structural (evidence-based) skill-testing within youth rugby. If so, please provide a specific reference. Mind the differentiation between a rise in the interest for this research topic on the one hand, and the actual use in (all-level) youth rugby practice on the other hand. For this manuscript, I would kindly request to be as precise as possible and as nuanced as necessary, as an alternative to broad blanket statements. Such demonstration of expertise will benefit its quality and ultimately the utility of your research

L47: Have been used (in research studies) to differentiate between playing level and identify talented players… Nuance.

L54: Too many spaces after “…between players...”?

L58: …reportedly compromised when tested among elite youth rugby league players.

L65: Skilled performance or skill performance?

L82: Suitable, as deemed by…the author? The authors of this manuscript? Arbitrarily deemed suitable?

L83-84: Please refrain from using non-specific terms such as “that recording” and “this approach”. Be specific with your references and thorough in your framing, as it is inappropriate to assume that all readers of your manuscript will have forefront knowledge of the content of the literature you refer to.

L84: “more appropriate…”, “more robust…”, for what purpose and as compared to what alternative? These wordings by themselves do not provide any contextual proof and therefore don’t hold a lot of meaning. Don’t just state this as fact. Why is this the best (more robust) method according to your expertise or the relevant literature?

L83-86: This composite sentence is too long and therefore becoming needlessly convoluted. Please rephrase with “one thought, one sentence” in mind. I suggest halving this sentence and starting again with “More importantly,…”

L86: Safety implications point already made prior; consider deleting this part.

L88: Insert reference to TackleTEC tool development protocol? Validation? Or at least link to the outcome version (Table 1).

L89-91: Please clarify if I understand correctly;

- The efficacy of this novel “TackleTEK programme” was shown for some specific outcomes, including tackle competency, as presented in the pilot data in reference [30].
- However, I have to assume tackling competency was not evaluated using an existing golden standard (these details are not available), but using the tackleTEK tool, which had not yet been validated or shown reliable.
- In addition to this, the programme was deemed effective for improving tackling skill based on this pilot data showing a median improvement measured in four (*arbitrary*?) of eighteen units, solely?

There seems to be an arbitrary nature to what is understood by effective improvement in overall tackling skill (4/18 units as measured by a non-validated tool). Furthermore, there seems to be a form of circular reasoning in which the outcome provided by this non-validated measuring tool is then used as a basis for a further post hoc reliability study of the exact measurement tool that was used to quantify the novel programme’s effectiveness. This is putting the cart before the horse.

L90: Four *arbitrarily* developed units? Four arbitrarily chosen units? Or do you mean progression on four randomly resulting units, out of 18 units? In this case, it is not *arbitrary*. Please clarify what you mean with “arbitrary”. If something was indeed developed or chosen arbitrarily, please explain why this is not an issue. Otherwise, it would be better to delete the word.

L91: Delete hyphen in five-weeks.

Additionally, please provide an up-to-date hyperlink to the actual book of abstracts with exact page ([2024_BOA_Web.pdf](https://www.ecss.mobi/DATA/CONGRESSES/GLASGOW_2024/DOCUMENTS/2024_BOA_Web.pdf) p. 647) within the reference list; this generic congress presentation reference does not lead to a readily accessible source containing the necessary information. Therefore, the reader cannot review the data presented to serve as evidence. Please mind that the burden of proof falls on you as the author.

L92: Please refrain from using generic wording such as “this”. Be specific; this what? What do you mean by considered “this” in relation to “analytical goal”? Please specify the units/items and clarify why these are suitable as the analytical goals in this research. Please be mindful, the reader does not hold the same insight as you, the researcher and author, regarding your research.

L96: “That is, …” replace semicolon with comma.

**METHODS**

**PARTICIPANTS**

L106: Delete comma after “written”.

L107: Consider additionally reporting the ranges for the descriptive characteristics of these participants, considering this is an age group at which large variances between subjects can exist, which may be more adequately be demonstrated with [min-max].

L108: Ideally, there would be a recognised classification of the rugby competence of the participating school boys, such as provide by the Participant Classification Framework by McKay and colleagues [1].

L110: Solely stating a two-and-a-half year period is too broad and rudimentary. Please provide more complete and structured information regarding the circumstances under which data collection was completed; specified periods, prescheduled collection design and/or recurrent collection dates, indoor/outdoor, pre/in/post-season, temperature and associated climatological conditions, time of day, and other conditions that may affect the outcome of the data collection. At this point there is no insight provided as to any considered or standardised approach of the data collection.

**DESIGN**

L112: Reliability…test?

L113: Familiarise participants ~~to~~ with… Additionally, remove the comma after tackles.

L112-114: Strange sentence structuring; please rewrite with “one thought, one sentence” in mind and aim to eliminate repeated wording, such as participants. For example: *“Participants were familiarised with the protocol through two tackles, limiting the learning effect. Each tackle…”*

L115: Replace “children” with participants. If a distinction needs to be made with the raters, prioritise using the term “players”. Please use this lexicon consistently throughout the manuscript.

L112-116: These phrases do not seem to add up. The authors state that the familiarisation consists of two tackles. Then the authors go on to state that these two tackles are across (so spread over) two visits (please reformulate to state “sessions”, as in test sessions or data collection sessions, rather than “visits”. These are no family matters). Following these statements, the authors contradict the previous statement by saying that the first “visit” is the familiarisation, and the second one is the actual rugby tackle assessment. Please clarify and rewrite this paragraph.

L116: Influenced in what way? Do you mean based on previous research? What was the(ir) original rationale? Did you copy-paste? Did you adapt and optimise? If so, in what way and why? Please provide an a priori rationale or some form of thorough explanation for why these “similar procedures” buttress the protocol used (in this different subpopulation). The rudimentary explanation at this point makes it seem as if the authors and/or coaching staff could have arbitrarily chosen a tackle protocol (i.e. familiarity; yet individual best-practice habits are not necessarily optimal), and post hoc retro-fitted this protocol with a variety of “similar procedures”, published from various rugby subpopulations. Please try to provide a more solid rationale evidencing an a priori evidence-based selection or development of the specific tackling protocol used in this study.

L118: As above, how was it updated. In what way?

L120: 10 by 10 = 10 x 10 m

L130: Missing details about the development of the TackleTEK tool (methodological process that resulted in the 18-point criteria?).

**FAMILIARISATION OF RATERS**

General:

A familiarisation session is good methodological practice in many cases, such as with the youth players for their tackle protocol execution. In this case, however, it is questionable whether a familiarisation session with the three raters is appropriate. If the use of the assessment tool requires extensive researcher-led familiarisation (including defining, explaining, and agreeing upon the criteria, as well as visual video assistance, demonstration of each individual criterion, and questioning), then it is not representable of, and therefore valid for use within youth rugby practice where this support will not be available. There is a considerable risk that the reliability testing will have been biased by the uniformity of understanding created in this familiarisation component.

L141: Please be precise and complete in your academic writing (use of commas, quotation marks, wording); i.e., “…which were labelled as “tier one”, “tier two”, and “tier three” for the current study, based…”

L143: As above. Alternatively, consider deleting “(tier one, two and three coaches…)”.

Remove first comma prior to “on the day…” (unnecessary).

“…before coding the first tackling assessment took place”= Strange sentence structure, please rephrase: “on the day of the first assessment, preceding the first tackling assessment”.

L144: explained,

L145: Replace “within” by “using”.

L148: In private? Or individually and in isolation from other raters and the (lead) researcher(s)?

**VIDEO SELECTION**

This paragraph comes across as a little confusing.

Were 25 tackle videos randomly selected from 206 original videos meeting the stated qualifying criteria? If this is correct, please restructure this passage somewhat to be more direct in conveying that core information. Or at least, try to frame the 25 random videos within the 206 videos, in one sentence, please. And delete redundancies.

L156: …videos,

L157: Define healthy, please? Do you mean not injured or *rugby (game or train) fit*? “Healthy” is too broad and generic a concept; it can mean whatever you want it to mean and is non-specific to rugby. Be precise, please.

L160: Which videos? The final 25 or the 206? Be precise, please.

L161: With or from 206 tackling videos?

**VIDEO EDITING**

L168: What is meant with “created for coding”? Were they created or randomly selected, or both? Are you referring to L155-157 “…25 rugby tackle videos of schoolboy participants were selected from pre-existing tackle assessment videos recorded as part of the TackleTEK Programme between 2022 and 2023”? Please keep in mind that the reader does not inherently possess the knowledge of the content of the TackleTEK programme. So, are you trying to say that the (more than) 206 videos from which the 25 final videos were randomly selected, were purposefully recorded with the single aim to be coded by the three rater in this study using the 18-point criteria? Please clarify.

L169: All (206) rugby tackle videos? Or the final selection of 25? Please clarify and be consistent and precise in your wording.

L174-176: The explanation the authors provide in this section leave it is unclear to me what or how the 25 videos were used for assessment. The 50 videos produced rugby tackle videos consisted of 25 tackle events but portrayed once from three camera angles and once from a single camera angle? What do you mean with “repeated twice”? The same event duplicated on video, i.e., viewed from different angles? The same players executing the “same” tackle twice? This writing is ambiguous.

L175: It seems methodologically inept to use duplicates of the same event to synthetically inflate your sample size. The influence of specific characteristics of such technically complex executions, especially when considered anomalous (outliers, if you will), could confound the outcome. This partly compromises the randomisation effect, and thus is suboptimal methodology. If the 25 + 25 videos were solely different in the camera angles used, both camera angles should have been available to the raters for the assessment of the same event. In addition, 25 other tackles could have been selected randomly to obtain a larger sample size.

**COACHES AND ASSESSMENT**

L178: The coaches coded the 50 tackle videos twice, once on each of two occasions, one week apart, correct? Please be precise.

L179: The possibility to watch and rewatch video footage is understandable within this observational research setup. However, it begs the question whether this procedure is transferable to real-world, and especially lower-level, youth rugby practice. It is certainly questionable whether it is pertinent to assume that such an involved procedure would be widely adopted in youth rugby, considering the technical and logistical (and financial) setup required on the one hand, and the time and energy investment for coaches on the other. This issue would be counter-productive to the aim of facilitating skill-based competency assessment in (youth) rugby practice, as stated in the introduction. In contrast, if this tool is meant to be used in direct observation (coach – player; no recording), then this research design is not valid for that purpose.

L183: Are worth… were worth (be consistent with the tense you are using within the paragraph and manuscript).

L184: How were coaches blinded from their own prior responses? Please elaborate.

**STATISTICAL ANALYSIS**

L194: These four points seem to nevertheless be fairly arbitrary.

L196-197 “(see [15])”, referring to Waldron et al. (2014) is not adequate. What point exactly with respect to the analysis of perfect agreement is it you want to make? This statement is methodologically lazy and leaves the reader guessing as to what the authors mean to pinpoint within this reference. Do Waldron and colleagues provide the argumentation for “why” to do this analysis in reliability studies? Is it a case of copy-paste? Please provide a concise yet solid guidance for the reader to understand your rationale, instead of assuming the reader will guess it by diving into underlying literature. It is your research; guide people through it, please.

L198-199: Can you specify which adjustments were made using the Bonferroni correction, please?

**CONCLUSION:**

L424: To my understanding, the analytical goals were arbitrarily established and are limited. As discussed above, these goals were distilled from the random outcome in the pilot study, using the very tool that is currently under investigation. The rigidity of this methodology is questionable and therefore it is problematic to use as base for strong conclusions.

L425: The use of the wording “can reach consistent levels of agreement…” is accurate indeed. Yet, it demonstrates my previous point, explicitly; based on the current research set up, which is questionable for broad extrapolation into youth rugby practice, one can select components that demonstrate reliability.

L426-431: I share the concerns you raise and commend your professional integrity to do so. As discussed before, the effect of coaching experience should have been accounted for as a potential confounder, within the study design. I understand the argument you present regarding the open nature of tackling skill and potential acquired knowledge of the experienced coach. At this point, this argument is logical, yet speculative, and it circumvents the issue at hand; in spite of the influence of the extensive familiarisation session, this small sample of raters did not show consistent reliability. It is thereby important to keep in mind the aim of developing a reliable tool for (wide-spread) use.

L432: Strength of the conclusions based on the limited analytical goals…?

L432-435: I cannot agree to the blanket statement that this tool can be used to assess tackling competency among schoolboys. This statement is factually inaccurate and somewhat misleading, perhaps evidencing (unconscious) investment bias or incentive-based bias. The tool demonstrated some promising features but the current design does not allow this tool to be labelled reliable in the overall context.

L436-437: I agree that more work is needed. The research presented is a good starting point but within the current research design certain structural limitation need to be addressed. A larger dataset within each rater tier-level would also provide for a stronger conclusion.
