# Supplementary material for: The Lions Sports Academy TackleTEK Tool: The intra-and inter-coach reliability of assessing tackling competency in rugby union
Source: PLoS One. 2026 Apr 24;21(4):e0346881. doi: 10.1371/journal.pone.0346881 (PMC13108810; doi:10.1371/journal.pone.0346881)
Supplement: Attachment — Submitted filename: Response to Reviewers.docx [file pone.0346881.s002.docx]

# Peer review

**GENERAL COMMENTS**

Dear authors, dear editor,

Thank you for the opportunity to review this manuscript. The development of youth rugby (skill) practice is an important aim within this research field, indeed. For this reason, I want to first and foremost commend the authors on their efforts to further the development of this research topic. I hope the sport can benefit from their continued contributions.

In the current study manuscript, there are various positive aspects. The overall intent to obtain a reliable measuring tool, examining both intra- and inter-rater reliability, is good practice. The statistical analysis is largely adequate and seems well-executed and reported. Conversely, the in-depth examination of the introduction, methods, and results section, have raised a number of serious concerns regarding the methodological design and development of this study. In addition, I have secondary concerns regarding the clarity with which certain sections of the manuscript are written. In conclusion, the information presented did not lead me to agree with the overall conclusion of this study.

The manuscript would benefit from greater methodological rigour. More attention should go towards precision and contextualisation of the information conveyed, such as pinpointing the particulars of the references used, as opposed to general lump-referencing or using blanket statements without precisely buttressing the argument for which the reference is integrated. The provision of more depth and appropriate nuance regarding the evidence used to frame the current research more precisely will facilitate the reader’s understanding of the current study and highlight the authors’ expertise. Lastly, greater attention should go towards correct and appropriate academic writing, regarding terminology, syntaxing, and the use of appropriate punctuation. These issues convolute the information presented. In my opinion, the manuscript should be restructured and rewritten to optimise the understanding of the actual protocol and its connection to and framing within the prior pilot study.

We thank the reviewer for their time and effort on our manuscript and providing us with a detailed review. The reviewer’s suggestions and challenges have helped us strengthen the manuscript throughout with justifications and explanations added through the introduction, methods, and results which support the findings that have been presented.

The main concerns regarding the methodological design and execution of this research study entail:

- The efficacy of the original pilot study (during which the tackling skill component of the TackleTEK programme was collected) was assessed using this non-validated tool, which is being investigated post hoc, in the current study, for its reliability.

Thank you for feedback but we would like to address the reviewer’s interpretation of the current work. To the best of our knowledge, despite numerous examples of tackling tests, there is no known validated tool or established process for validating a tool to identify tackling-related skills. There are a number of tests that have established the reliability (in various ways) of their test and frequently report an error statistic (den Hollander et al., 2019; Gabbett, 2008), but without development of analytical goals. Our approach is in-keeping with the notion of *a-priori* analytical goals (Atkinson & Nevill, 1998), which we have determined based on pilot data, as suggested by the authors of the seminal work (Atkison & Nevill, 1998). Subsequently, we have applied the non-parametric 95% limits of agreement method (Cooper et al., 2007) and adopted the *a-priori* analytical goal to evaluate the reliability in a meaningful way. This provides readers with an understanding of the error of the test in the context of a realistic analytical goal. We also provide an assessment based on ‘perfect agreement’ to enable the reader to understand the importance of interpreting error (and its acceptability) in cases where the analytical goal may change. This important addition to the literature has not featured in the interpretation of the reviewer, and we ask that further thought is given to the above information.

- Based on this original pilot data for which this tool was used, the progress shown in four of eighteen ‘units’ was, seemingly arbitrarily, deemed adequate as the analytical goals for the current study. Apart from the arbitrary nature, I fear these first two points demonstrate a form of circular reasoning.

Thank you for your feedback and the concern you raise of using pilot data. As above, an established framework has been followed that emphasises the setting of clear evidence-based analytical goals (Atkinson & Nevill, 1998). In the current study, four of eighteen units equated to the median improvement of children in the composite tackling score and provided a means to understand whether the potential errors within and between raters would enable the detection of changes of this magnitude. Therefore, it is quite the opposite to the arbitrary selection referred to above by the reviewer, and we direct them to the papers cited in our responses and the current paper, which states where the goal originated and how it was selected. There is no circular reasoning.

- Although the participants were defined as being the youth players, as this is a reliability study, the raters are arguably the subjects under investigation. The number of raters (3) is limited. In addition, these three raters differ greatly in their perceived coaching expertise, their only reported differentiating characteristic. This heterogeneity in experience level was however not accounted for as a confounder, and practically dismissed in the conclusion with a caveat. I would have liked to see a greater number of raters within each of the three tier-levels to account for this effect and strengthen this study’s generalisability.

We thank you for your feedback and we agree that this point is arguable. The participants are the youth players, as they were recruited with informed consent and received the instruction of how to perform the standardised tackling protocol, with the raters deemed to be part of the assessment tool. The generalisability of the tool was not being assessed, and the number of raters was determined based upon current research, in which two or three raters are often used to determine the reliability of tackling assessments (den Hollander et al., 2019; Waldron et al., 2014; Gabbett, 2008).

The inclusion of coaches of different coaching levels was intentional and part of the research question, as stated in the manuscript. By definition, a cofounder is a factor that has an influence on the measured variables, and the fact we measured the effects of this by comparing the tool within and between raters demonstrates that this wasn’t overlooked – it was a main feature of the study.

- Using duplicates of video footage samples (25x2) to inflate the sample size (50), even though from a different angle, is a questionable practice. Especially considering the availability of sufficient original cases (206).

We are not sure on what basis this is questionable. As the reviewer alluded to above, part of this study is to assess the new test’s (and the raters of the test) ability to rate tackling competency. The interpretation of the rater when using the tool is under investigation and the conditions are the same for all raters. To test the reliability of their scoring whilst incorporating different views/camera angles, this approach is acceptable. Indeed, this provided an opportunity to test rater scoring under altered conditions, which may even strengthen the design.

Our dataset appears to be more than sufficient, given that research in this area has utilised as few as five tackles, by five players, scored from a single angle from video footage (den Hollander et al., 2019) to 20 players who repeated four to six tackles to be scored from single angle (Waldron et al., 2014).

- There is a complete lack of detail regarding the context in which the original player data was collected during more than two and a half years. Consequently, there is no way to assess the in-practice standardisation of the protocol.

Thank you for your feedback. The standardisation in practice is achieved through the clear scoring against pre-defined criteria and a structured assessment process, rather than the broader context of the data collection (Hopkins, 2000; Atkinson & Nevill, 1998). That said, we have added the time course in which the data was collected, L121-122: “All data collection was performed during the school term from September to April outdoors, between the hours of 2-4 pm, as part of regular games lessons”, to the manuscript to demonstrate the controlled manner in which the data were collected in a real-world school rugby setting. As the aim of the study was to assess the inter- and intra-reliability, the emphasis is on the robustness of the rating tool itself, not the variability of the external conditions across time. I.e. the videos are the same within and between raters. Therefore, though the protocol was clearly conducted in a controlled manner, the conditions for the raters was consistent and this is what matters. We trust that the explicit description in the methods makes the study easily replicable.

- The extensive familiarisation process undertaken with the raters by the lead researcher raises the question to what degree the outcome was influences by explicitly making the interpretation of the tool’s criteria as uniform as possible; a quality that would be inherent to a valid and reliable measuring tool. The concern is that its ability to be extrapolated to in-practice youth rugby is therefore compromised.

Thank you for your feedback regarding the efficacy of the familiarisation process. Rigorous familiarisation procedures are necessary to support the development of robust methods and to improve reliability measurements, which should also be replicated in the field (Hopkins, 2000). Had we not included any familiarisation, then there would be many more criticisms and risks to the study. We are happy that this degree of familiarisation was necessary and view it as a strength of the study.

- The choice to use multi-angle video footage (with ad libitum rewatch) instead of live assessment of tackling skill also begs the question to which degree these outcomes are generalisable to youth rugby practice, at all levels, which to my understanding is the ultimate aim.

Thank you for your feedback and concern regarding the use of the tool performed with ad libitum rewatch and it’s use across grassroots rugby union. Important decisions on tackling competency (a very complex skill, involving two people) are extremely challenging in a live environment (Waldron et al., 2014; Gabbett, 2008). This approach allowed for raters to provide a repeatable and comprehensive observational process, controlling potential sources of variability in the live environment, which can affect the measurement of reliability. Additionally, the use of ad libitum rewatching at variable speeds enables raters to review ambiguous moments within a tackle multiple times to reduce the likelihood of errors through misinterpretation, which can occur in live assessments (Spitz et al., 2018). The use of recorded footage is a more responsible way for coaches to focus on the young players performing tackles. If we had chosen live assessments only, there would be many more criticisms of this approach.

- Data restrictions apply and it seems anonymised data is also not available. Therefore, these already limited results are not reproducible.

The reviewer can fully replicate the methods of this study and does not need access to results to do so. As per the ethical approval of this research, the raw results are unavailable. The transparent methodological approach can be followed by others and their own evidence-based analytical goals can be determined.

Regarding the conclusion of this study, within its design, the results show select aspects of reliability, indeed. Notwithstanding, I do not agree that the information presented unambiguously shows that the tackleTEK tool is an overall reliable method for assessing changes in tackling competency. This is largely based on the questionable methodological design issues, as outlined above.

We thank you for your feedback; however, based on our responses above, we trust that the reviewer can understand that the link to real-world and context-specific analytical goals has been made. It would be useful in the future to apply the tool to other scenarios where the goals are different, and this would increase the generalizability of its use. The overriding message here is that there is no such ‘black and white’ or ‘yes and no’ conclusion for the reliability of a measurement/test, as this clearly depends upon the use-case, and to conclude anything outside of what we have would clearly be erroneous, despite many other researchers doing so. We have stated this in a future research section of the discussion (L455-457).

L455-457: “Applying the TackleTEK Tool across other playing levels, with appropriate analytical goals, would increase the general use of the tool throughout rugby union.”

I believe this study may be a worthwhile addition to a thesis, if its contextual limitations are appropriately framed within the overarching research. However, for the reasons discussed, I am afraid I cannot recommend this manuscript for publication in PLOS ONE.

We thank you for your candid feedback regarding the manuscript; however, we hope that our responses allay your concerns, whilst demonstrating the importance of this work.

Please find further review details below.

**ABSTRACT**

Line 25-26: Strangely worded: “...however, it is important to establish the reliability of these tests to understand the capacity to identify change across time.”

Reworded to clarify that previous tackling assessments have focused on talent identification and not been tested specifically for the purpose of monitoring skill development across multiple time points.

L23-25: “Tackling skill tests in rugby aim to evaluate skill competency, but their reliability to detect meaningful changes in tackling competency over time, that align to real-world analytical goals, remains uncertain.

This specific test or all such tests? The tool’s capacity to identify change? Change of what? Be specific, please. I believe you mean to say, you want to be able to confidently (reliably) identify change in tackling competency across time, using this specific test. Therefore, the testing tool needs to be reliable, correct? For instance, an unreliable tool would perhaps be more prone to “identify change”, by its very nature. So too could an invalid testing tool, by not measuring the variable of interest effectively. Please rephrase this sentence.

L23-25 Reworded to clarify the with specific wording to reduce ambiguity.

Line 29-30: “Analytical goals were developed…” This sentence is a filler with no added benefit to understanding the design, as it does not hold any useful information at this point in the manuscript; what analytical goals, why, how were they developed, and by whom? Please frame it so that its importance becomes apparent. Or alternatively leave it out if redundant.

Sentence added to show an objective goal was formed from pilot data using a rigorous iterative process, which is necessary to be able to assess reliability.

L28-309 “Pilot data was used to objectively develop *a-priori* analytical goals necessary for assessing the reliability of composite and subcomponent scoring.”

Line 30: Subcomponent, no hyphen, is the more common spelling. Both allowed, though.

The hyphen has been removed throughout the manusctipt for the word subcomponent.

Line 33: Tacking missing an “l”.

This has been corrected.

L33: “…tackling…”.

Line 34-35: “the analytical goal of ≤4 points” at this point in the manuscript does not hold much meaning to the reader, as it has not been explained or framed. This statement assumes inside information is present before the readers gets to the body of the article. Please provide some clarification and/or rephrase.

L28-29 and L34-35 have been amended.

L28-29: “Pilot data was used to objectively develop *a-priori* analytical goals necessary for assessing the reliability of composite and subcomponent scoring.”

Line 37-38: “Intra-coach sub-component analysis showed the tier one and tier two coach to repeatably score all sub-components (P > 0.05); however,…”. So, all subcomponents were scored reliably alike, or consistently reliable, yet non-significantly so? What are you trying to convey exactly? “Repeatably” is not the optimal way to infer reliability. First off, it sounds as if the act of scoring subcomponents by coaches itself was the measurement of interest, not the reliability of the scores. Additionally, provide an explanation for the p-value. Please rephrase.

All hyphens have been removed from the word subcomponent. The scoring of subcomponents is of interest and has been identify as the second study aim. On L32-33 the addition of “. Systematic differences were assessed using Wilcoxon tests” to explain the *P*-value. The coaches must repeat the test twice as the error between the two testing sessions is what is assessed for the systematic differences against the analytical goal. If preferred the term reliable can be used in place of repeatability.

**INTRODUCTION**

General note:

There is a need to define, early on, what the authors mean by the prevalently used terminology of “(sub-component) analytical goals”. What exactly do you mean by this wording within your research? Further to that, terminology such as “sub-components” and “arbitrary units” could be framed more clearly. Clarifying such terminology will greatly facilitate the readers understanding throughout, from the start. Assume that the reader does not know any of the research you aim to share.

The term subcomponent has been defined on lines L94-97. As tackling tests lack standardised units of measurement (SI units) they were reported in arbitrary units (AU), ais commonly used to standard measurement, across all sciences, when recognised measurement units are unavailable. To evaluate the acceptability of the measurement error, a real-world target defined (i.e., an analytical goal), based on the approach detailed by Atkinson and Nevill (1998). This approach acknowledges that reliability should not be assessed in binary terms (i.e., reliable or unreliable), but rather to understand whether the inevitable error of measurement is acceptable against a real-world threshold. The analytical goal provides a context-specific threshold to determine whether the observed error is meaningful to be applied in its specific setting.

L94-97: “To ensure the goal for assessing reliability is robust, Atkinson and Nevill [30] recommend the implementation of a goal drawn from empirical evidence (pilot data), as it provides an objective measure from the observed phenomena to refine the allocation of a realistic, real-world goal, mitigating the application of a subjective measure.”

L46-47: This is an inaccurate representation of the literature referenced, or at least lacking in nuance; skill-testing is described as a useful tool for differentiation and ID identification purposes. The literature surrounding this broad topic (in various sports, not solely rugby union) has steadily increased, indeed. Consequently, so have the protocols and frameworks provided. However, the implementation of these protocols and frameworks are not commonplace and are mainly reserved for talented youth programmes. To my knowledge, no study has investigated the implementation, i.e., the prevalence of structural (evidence-based) skill-testing within youth rugby. If so, please provide a specific reference. Mind the differentiation between a rise in the interest for this research topic on the one hand, and the actual use in (all-level) youth rugby practice on the other hand. For this manuscript, I would kindly request to be as precise as possible and as nuanced as necessary, as an alternative to broad blanket statements. Such demonstration of expertise will benefit its quality and ultimately the utility of your research

Thank you for your feedback, we agree that the abundance of the assessments are predominantly used within elite or player pathway programmes, L45 has been amended to reflect this.

L45: “Skill testing among youth team athletes is common practice in talented player pathways [1-3].”

L47: Have been used (in research studies) to differentiate between playing level and identify talented players… Nuance.

Taking on your feedback L46-48 has been amended to provide more nuance to the specific use of whether the results are used in practice or research.

L46-48: “Results from these tests are currently used, in research, to differentiate between playing levels [1,4,5] or, in practice, to establish an athlete’s competency in executing a sport-specific skill [1,6,7].”

L54: Too many spaces after “…between players...”?

Spacing between words has been checked and corrected.L53: “…between players and…”

L58: …reportedly compromised when tested among elite youth rugby league players.

Sentence amended to frame the participants of the study.

L57: “…compromised in the assessment of elite youth rugby league players…”

L65: Skilled performance or skill performance?

This has been edited to clarify that player safety is reliant on the execution of the tackling skill.

L62: “…reliant upon the correct tackling skill execution” to show it relates to ‘skill performance’.

L82: Suitable, as deemed by…the author? The authors of this manuscript? Arbitrarily deemed suitable?

This has been edited to show that the analytical goals were created following rigorous methods detailed by Atkinson and Nevill (1998), as evidenced earlier within this rebuttal. This has made the Atkinson reference switch position in the reference list, and the references have been amended accordingly throughout the doc.

L80-81: “…and analytical goals were targeted using methods detailed by Atkinson & Nevill [30]”

L83-84: Please refrain from using non-specific terms such as “that recording” and “this approach”. Be specific with your references and thorough in your framing, as it is inappropriate to assume that all readers of your manuscript will have forefront knowledge of the content of the literature you refer to.

Thank you for your feedback to address the lack of specificity the mention lines have been adjusted accordingly. L82 Reworded “…recorded during…” to “…video data recorded during…”. L82 Reworded “this approach” to “…using NP 95% LOA…” which has been defined earlier in the manuscript.

L84: “more appropriate…”, “more robust…”, for what purpose and as compared to what alternative? These wordings by themselves do not provide any contextual proof and therefore don’t hold a lot of meaning. Don’t just state this as fact. Why is this the best (more robust) method according to your expertise or the relevant literature?

The non-parametric 95% limits of agreement (NP 95% LOA) method is more appropriate with this dataset, than methods such as the intraclass correlation coefficient (ICC). The NP 95% LOA method quantifies both systematic bias and random variability by showing the range within 95% of the differences of repeated tests usually occur. This allows results to be evaluated against a real-world, context-specific, analytical goals (Bland & Altman, 1999), thus, the error can then be interpreted to determine whether it is small enough to be acceptable for real-world use (Bland & Altman, 1999). In contrast, ICC provides a correlation coefficient which provides a number and the idea of relative reliability making goal setting based on known disagreement levels more challenging (Atkinson & Nevill, 1998). This has been edited into the manuscript.

L81-89: “For data on nominal or ordinal scales, such as video data recorded during coaching assessments, using NP 95% LOA is more appropriate, but more importantly, offers a more robust statistical method with parametric datasets, like differentiating tackling competency levels. The NP 95% LOA method quantifies measurement error, capturing both systematic and random error, and aligns with context-specific analytical goals, unlike methods such as intraclass correlation coefficient [30]. It also shows the range within 95% of the differences occur between repeated tests. These results can then be compared against predefined thresholds, i.e., context-specific analytical goals, to interpret whether the measurement error is acceptable for real-word use [30].”

L83-86: This composite sentence is too long and therefore becoming needlessly convoluted. Please rephrase with “one thought, one sentence” in mind. I suggest halving this sentence and starting again with “More importantly,…”

Thank you for the feedback the rewriting of the previous comment resolved the concern by splitting the sentence with the comparison of another reliability method, which is less appropriate for this specific assessment.

L86: Safety implications point already made prior; consider deleting this part.

The implications for safety have been removed.

L88: Insert reference to TackleTEC tool development protocol? Validation? Or at least link to the outcome version (Table 1).

L90-93 Sentence inserted to specify the co-development of the standardised protocol and criteria as per previous research by Gabbett (2008) and Waldron et al. (2014).

L89-91: Please clarify if I understand correctly;

- The efficacy of this novel “TackleTEK programme” was shown for some specific outcomes, including tackle competency, as presented in the pilot data in reference [30].

Tackling competency was assessed using the TackleTEK Tool and reported upon in this reference to a published conference abstract. These data provide the ideal opportunity to develop context-specific analytical goals.

- However, I have to assume tackling competency was not evaluated using an existing golden standard (these details are not available), but using the tackleTEK tool, which had not yet been validated or shown reliable.

The notion of something being reliable or not (yes or no) is not possible, unless a measurement tool is totally free of error, which seldom occurs. Please see Atkinson and Nevill (1998). We hope we have made this point clear. This is the paper where we assess its reliability but do so more comprehensively based on real-world data. There is no gold-standard assessment for measuring changes in tackling competency. We do also present results of an analytical goal of ‘perfect agreement’, to offer the reader the understanding of how changing the analytical goal would alter the interpretation.

In addition to this, the programme was deemed effective for improving tackling skill based on this pilot data showing a median improvement measured in four (*arbitrary*?) of eighteen units, solely?

The pilot data we are reporting on, demonstrated a 4-point median increase in tackling skill in an intervention group, which was statistically significant as detailed in L94-102. The units of measurement have no recognised SI unit and are therefore AU best described as AU. Which has been clarified with an addition to lines 99-100.

There seems to be an arbitrary nature to what is understood by effective improvement in overall tackling skill (4/18 units as measured by a non-validated tool). Furthermore, there seems to be a form of circular reasoning in which the outcome provided by this non-validated measuring tool is then used as a basis for a further post hoc reliability study of the exact measurement tool that was used to quantify the novel programme’s effectiveness. This is putting the cart before the horse.

As per our previous responses, there is no circular reasoning here. As detailed by Atkinson and Nevill (1998) and Cooper et al. (2007), the methods adopted within this study assess reliability and interpret this in the context of an *a-priori* analytical goal. There is no validated tool for this purpose, and we have not claimed to assess this here.

L90: Four *arbitrarily* developed units? Four arbitrarily chosen units? Or do you mean progression on four randomly resulting units, out of 18 units? In this case, it is not *arbitrary*. Please clarify what you mean with “arbitrary”. If something was indeed developed or chosen arbitrarily, please explain why this is not an issue. Otherwise, it would be better to delete the word.

The term arbitrary was used as a classification of a measuring unit, to reduce the chances of misinterpretation of the reader the word arbitrary can removed from the manuscript. To be clear, there is nothing arbitrary about the selection of the four units.

L97: Delete hyphen in five-weeks.

The hyphen has been deleted.

Additionally, please provide an up-to-date hyperlink to the actual book of abstracts with exact page ([2024_BOA_Web.pdf](https://www.ecss.mobi/DATA/CONGRESSES/GLASGOW_2024/DOCUMENTS/2024_BOA_Web.pdf) p. 647) within the reference list; this generic congress presentation reference does not lead to a readily accessible source containing the necessary information. Therefore, the reader cannot review the data presented to serve as evidence. Please mind that the burden of proof falls on you as the author.

Thank you for your feedback. The hyperlink has been added to the reference list, which takes the reader to the exact location within the book of abstracts. We accept the burden of proof for the information stated, as it was presented at an international conference. Access to the presentation slides is available on contacting the authors.

L92: Please refrain from using generic wording such as “this”. Be specific; this what? What do you mean by considered “this” in relation to “analytical goal”? Please specify the units/items and clarify why these are suitable as the analytical goals in this research. Please be mindful, the reader does not hold the same insight as you, the researcher and author, regarding your research.

L99 The wording of the sentence has been amended to be specific to the goal that was determined by following methods detailed by Atkinson and Nevill (1998). We feel that the meaning and reference to the meaning is very clear. Our articulation of the question is consistent with our previous publications in this area across the last few decades and is sufficient to be interpreted correctly.

L96: “That is, …” replace semicolon with comma.

Thanks for your feedback, this has been changed on both L108 and L345.

**METHODS**

**PARTICIPANTS**

L106: Delete comma after “written”.

Comma deleted.

L107: Consider additionally reporting the ranges for the descriptive characteristics of these participants, considering this is an age group at which large variances between subjects can exist, which may be more adequately be demonstrated with [min-max].

Thank you for your feedback, L116-117 have been modified to represent ranges of the participant characteristics.

L108: Ideally, there would be a recognised classification of the rugby competence of the participating school boys, such as provide by the Participant Classification Framework by McKay and colleagues [1]. L 1114-118 More detail has been provided about the provision of rugby coached offered to the participants; however, the Participant Classification Framework by Mcay et al. (2021) is conceptualised for adults from the age of 18 to 64 years and so not appropriate to apply to the cohort represented within this manuscript.

L110: Solely stating a two-and-a-half year period is too broad and rudimentary. Please provide more complete and structured information regarding the circumstances under which data collection was completed; specified periods, prescheduled collection design and/or recurrent collection dates, indoor/outdoor, pre/in/post-season, temperature and associated climatological conditions, time of day, and other conditions that may affect the outcome of the data collection. At this point there is no insight provided as to any considered or standardised approach of the data collection.

Thank you for your feedback, L121-122 now provide greater detail in the data collection conditions.

L121-122: “All data collection was performed in the rugby playing school terms from September to April outdoors, between the hours of 2-4 pm, as part of regular games lessons.”

**DESIGN**

L112: Reliability…test?

This has been updated to read L126 “…reliability assessment…”.

L113: Familiarise participants ~~to~~ with… Additionally, remove the comma after tackles.

L127-129 Sentences have been rewritten for reader clarification.

L112-114: Strange sentence structuring; please rewrite with “one thought, one sentence” in mind and aim to eliminate repeated wording, such as participants. For example: *“Participants were familiarised with the protocol through two tackles, limiting the learning effect. Each tackle…”*

L127-129 have been rewritten.

L115: Replace “children” with participants. If a distinction needs to be made with the raters, prioritise using the term “players”. Please use this lexicon consistently throughout the manuscript.

As per the recommendation, through the remainder of the manuscript, the word ‘children’ has been replaced where appropriate to the term participants. It is important to appreciate that these players are not solely rugby players competing for performance target but rather are children playing in a participation sport for which the tool is purposely designed for.

L112-116: These phrases do not seem to add up. The authors state that the familiarisation consists of two tackles. Then the authors go on to state that these two tackles are across (so spread over) two visits (please reformulate to state “sessions”, as in test sessions or data collection sessions, rather than “visits”. These are no family matters). Following these statements, the authors contradict the previous statement by saying that the first “visit” is the familiarisation, and the second one is the actual rugby tackle assessment. Please clarify and rewrite this paragraph.

Thank you for your feedback, L127-129 have been rewritten to clarify the procedure.

L116: Influenced in what way? Do you mean based on previous research? What was the(ir) original rationale? Did you copy-paste? Did you adapt and optimise? If so, in what way and why? Please provide an a priori rationale or some form of thorough explanation for why these “similar procedures” buttress the protocol used (in this different subpopulation). The rudimentary explanation at this point makes it seem as if the authors and/or coaching staff could have arbitrarily chosen a tackle protocol (i.e. familiarity; yet individual best-practice habits are not necessarily optimal), and post hoc retro-fitted this protocol with a variety of “similar procedures”, published from various rugby subpopulations. Please try to provide a more solid rationale evidencing an a priori evidence-based selection or development of the specific tackling protocol used in this study.

Thank you for your feedback. L130-132 has been rewritten. Now the information portrays how the assessment protocol and tackling criteria influenced the creation of the TackleTEK Tool. Many of the criteria from previous research overlap as the key components to tackling have long been established; however, the criteria have been sub-divided into differing components and additional criteria were incorporated from tackling biomechanics research published after the previous tackling tools.

L118: As above, how was it updated. In what way?

This has been incorporated within the rewrite of the previous feedback.

L120: 10 by 10 = 10 x 10 m

L133 has been changed to 10 x 10 m

L130: Missing details about the development of the TackleTEK tool (methodological process that resulted in the 18-point criteria?).

Thank you for your feedback, the method of criteria development has been elaborated on L141-146.

**FAMILIARISATION OF RATERS**

General:

A familiarisation session is good methodological practice in many cases, such as with the youth players for their tackle protocol execution. In this case, however, it is questionable whether a familiarisation session with the three raters is appropriate. If the use of the assessment tool requires extensive researcher-led familiarisation (including defining, explaining, and agreeing upon the criteria, as well as visual video assistance, demonstration of each individual criterion, and questioning), then it is not representable of, and therefore valid for use within youth rugby practice where this support will not be available. There is a considerable risk that the reliability testing will have been biased by the uniformity of understanding created in this familiarisation component.

Thank you for your feedback. Familiarisation is important in this setting, owing to the complex nature of the tackling skill and the human observers benefit of clear, shared understanding before scoring. Research, from clinical health care sciences and sports science, recommend familiarisation sessions for raters to achieve acceptable levels of reliability (Wilke et al., 2023), by providing clear instruction of the video coding process which reduces the variability in scoring through personal interpretation of the working definitions and the minimises the effects of prior experience, especially when assessing nursing and health care contexts (Haidet et al., 2009). Additionally, the use of practice clips is promoted to ensure raters have a clear understanding of the criteria and scoring system, for instance when assessing pharmacists’ behavioural skills with clients (Smith et al., 2024). An observer-based tool to assess physical literacy in games-based assessments, in physical education, was only shown to be highly reliable as a tool following a detailed observer training and familiarisation protocol (Wilke et al., 2023). There is no reason this process should change here, when rating a complex skill, that when performed in line with a technical model can reduce injury risk in youth sport. Furthermore, the current study utilised coaches who were uninvolved in the criterion development phase to mitigate the potential bias.

L141: Please be precise and complete in your academic writing (use of commas, quotation marks, wording); i.e., “…which were labelled as “tier one”, “tier two”, and “tier three” for the current study, based…”

Thank you for the feedback, grammatical changes have been made to include single line quote marks which are appropriate for highlighting labels.

L158-159: “…which were labelled as ‘tier one’, ‘tier two’, and ‘tier three’ for the purposes of the current study, based…”

L143: As above. Alternatively, consider deleting “(tier one, two and three coaches…)”.

Deleted as advised.

Remove first comma prior to “on the day…” (unnecessary).

Comma deleted as advised.

“…before coding the first tackling assessment took place”= Strange sentence structure, please rephrase: “on the day of the first assessment, preceding the first tackling assessment”.

Thank you for your feedback, L159-161 has been rewritten for reader clarity.

L144: explained,

Comma added.

L145: Replace “within” by “using”.

Thank you for the feedback, the word ‘within’ has been replaced with ‘using’.

L148: In private? Or individually and in isolation from other raters and the (lead) researcher(s)?

L164-165 The word ‘private’ has been replaced with ‘isolation’ and rewritten to describe how the coaches’ questions were privately conducted and how the first assessment was completed.

**VIDEO SELECTION**

This paragraph comes across as a little confusing.

Were 25 tackle videos randomly selected from 206 original videos meeting the stated qualifying criteria? If this is correct, please restructure this passage somewhat to be more direct in conveying that core information. Or at least, try to frame the 25 random videos within the 206 videos, in one sentence, please. And delete redundancies.

Thank you for your feedback, L172-180 have been rephased.

L156: …videos,

This has been absorbed into the rephrasing of L172-180.

L157: Define healthy, please? Do you mean not injured or *rugby (game or train) fit*? “Healthy” is too broad and generic a concept; it can mean whatever you want it to mean and is non-specific to rugby. Be precise, please.

Thank you for the feedback, this has been specified within the rephasing of this section through L174.

L160: Which videos? The final 25 or the 206? Be precise, please.

Rephrased to specify on L179 “…the final 25 video recordings…”.

L161: With or from 206 tackling videos?

L176-180 has been rephrased for clarity of the video selection process.

**VIDEO EDITING**

L168: What is meant with “created for coding”? Were they created or randomly selected, or both? Are you referring to L155-157 “…25 rugby tackle videos of schoolboy participants were selected from pre-existing tackle assessment videos recorded as part of the TackleTEK Programme between 2022 and 2023”? Please keep in mind that the reader does not inherently possess the knowledge of the content of the TackleTEK programme. So, are you trying to say that the (more than) 206 videos from which the 25 final videos were randomly selected, were purposefully recorded with the single aim to be coded by the three rater in this study using the 18-point criteria? Please clarify.

Thank you for your feedback. To provide more concise and precise information of the protocol the whole section (L185-193) has been reworded. The section now clearly refers to the final 25 randomly selected tackling videos.

L169: All (206) rugby tackle videos? Or the final selection of 25? Please clarify and be consistent and precise in your wording.

As above, the section has been reworded to be more precise with the wording and concise with the methods.

L174-176: The explanation the authors provide in this section leave it is unclear to me what or how the 25 videos were used for assessment. The 50 videos produced rugby tackle videos consisted of 25 tackle events but portrayed once from three camera angles and once from a single camera angle? What do you mean with “repeated twice”? The same event duplicated on video, i.e., viewed from different angles? The same players executing the “same” tackle twice? This writing is ambiguous.

The rewording of this section has specific the use of the differing camera angles to reduce the ambiguity of how the total of 50 tackling videos that were used.

L175: It seems methodologically inept to use duplicates of the same event to synthetically inflate your sample size. The influence of specific characteristics of such technically complex executions, especially when considered anomalous (outliers, if you will), could confound the outcome. This partly compromises the randomisation effect, and thus is suboptimal methodology. If the 25 + 25 videos were solely different in the camera angles used, both camera angles should have been available to the raters for the assessment of the same event. In addition, 25 other tackles could have been selected randomly to obtain a larger sample size.

Thank you for your feedback and expressing your concerns over the use and sample size of the videos for analysis. We did not attempt to inflate the sample size and had no need to do this. In the most recent research, tackling competency tools have investigated reliability using 20 tackle actions of 5 participants from a selection of 655 tackles from 131 participants (den Hollander et al., 2019), demonstrating that the sample used within the current study is large enough to determine if the tool is acceptable for use. Importantly, the raters use of the tool is being analysed, and the differing camera angles offer another view from which the raters can judge tackling competency.

**COACHES AND ASSESSMENT**

L178: The coaches coded the 50 tackle videos twice, once on each of two occasions, one week apart, correct? Please be precise.

L190-193 Reworded to be specific with the assessment protocol as recommended.

L179: The possibility to watch and rewatch video footage is understandable within this observational research setup. However, it begs the question whether this procedure is transferable to real-world, and especially lower-level, youth rugby practice. It is certainly questionable whether it is pertinent to assume that such an involved procedure would be widely adopted in youth rugby, considering the technical and logistical (and financial) setup required on the one hand, and the time and energy investment for coaches on the other. This issue would be counter-productive to the aim of facilitating skill-based competency assessment in (youth) rugby practice, as stated in the introduction. In contrast, if this tool is meant to be used in direct observation (coach – player; no recording), then this research design is not valid for that purpose.

Thank you for your concern about the real-world application of the TackleTEK Tool. Video analysis of fast, complex skills, i.e., tackling, offers frame-by-frame review to improve objectivity, exceeding the fidelity of live observation, for accurate evaluation of movement quality and competency (Shill et al., 2023). This is highly important for collision skills, like tackling, where judgements can improve technical ability and importantly minimise injury risk in a sport under severe scrutiny from government advisors (Pollock et al., 2017). This protocol is already in use within grassroots rugby, with Lions Sports Academy having worked with hundreds of players across schools and clubs, where the videos are available to players. Filming is more financially available to everyone as high-quality video footage can be captured on a phone if needed.

L183: Are worth… were worth (be consistent with the tense you are using within the paragraph and manuscript).

Thank you for your feedback, changes have been made through L194-203 to ensure the tense is consistent throughout the manuscript.

L184: How were coaches blinded from their own prior responses? Please elaborate.

Thank you for your feedback, to clarify how coaches were blinded to previous responses an addition to L201-202 of “…(unable to review previously scored tackle ratings),…” was included.

**STATISTICAL ANALYSIS**

L194: These four points seem to nevertheless be fairly arbitrary.

The four points were based upon pilot work, where statistical differences were found and reported. This is the recommended process for developing an analytical goal, to support that thresholds are based on context-specific evidence rather subjective judgement (Cooper et al., 2007; Atkinson & Nevill, 1998).

L196-197 “(see [15])”, referring to Waldron et al. (2014) is not adequate. What point exactly with respect to the analysis of perfect agreement is it you want to make? This statement is methodologically lazy and leaves the reader guessing as to what the authors mean to pinpoint within this reference. Do Waldron and colleagues provide the argumentation for “why” to do this analysis in reliability studies? Is it a case of copy-paste? Please provide a concise yet solid guidance for the reader to understand your rationale, instead of assuming the reader will guess it by diving into underlying literature. It is your research; guide people through it, please.

L214-215 has been reworded to show that the perfect agreement analysis was also conducted to demonstrate the use of a scenario where perfect agreement (no error) was required. This is useful to understand how different analytical goals would affect the interpretation of the tool’s reliability, which may be the case if perfect agreement was required. We reference Waldron et al. (2014), as the same process was undertaken.

L198-199: Can you specify which adjustments were made using the Bonferroni correction, please?

Thank you for your feedback. Additional information has been written into L218-221. This includes the calculation of the adjustments, and the and the adjusted alpha level.

**CONCLUSION:**

L424: To my understanding, the analytical goals were arbitrarily established and are limited. As discussed above, these goals were distilled from the random outcome in the pilot study, using the very tool that is currently under investigation. The rigidity of this methodology is questionable and therefore it is problematic to use as base for strong conclusions.

Thank you for your feedback regarding the development of the *a-priori* analytical goals; however, the analytical goals were not ‘arbitrarily established’, are far from ‘random’ and are not ‘limited’. These goals were established based on pilot data, which is the approach advised by seminal reviews in this field (Hopkins, 2000). Pilot data serves to develop an analytical goal for the tool. How else would one develop a context-specific analytical goal? To our experience and knowledge, there is no better approach. Other options include determining ICCs and concluding with the ICC being ‘moderate’ or ‘strong’ etc. What use is this to researchers or coaches in the field?

Additionally, the assertion that the methodology is "rigid" and "problematic" fails to acknowledge the flexibility of the approach, which lends itself to any analytical goal that the user requires. This is the opposite to other redundant approaches to reliability, which typically provide a fixed coefficient value.

L425: The use of the wording “can reach consistent levels of agreement…” is accurate indeed. Yet, it demonstrates my previous point, explicitly; based on the current research set up, which is questionable for broad extrapolation into youth rugby practice, one can select components that demonstrate reliability.

We encourage the reviewer to open their mind to the process of reliability interpretation that we have adopted here and is consistent with expert recommendations (Cooper et al., 2007; Atkinson and Nevill, 1998), as well as being logical. We have taken steps to understand the error of a tool against an evidence based analytical goal. Others can also. The reviewer appears to be looking for something that doesn’t exist. It is logical that components of the test can be reliable for this current purpose but not for others.

L426-431: I share the concerns you raise and commend your professional integrity to do so. As discussed before, the effect of coaching experience should have been accounted for as a potential confounder, within the study design. I understand the argument you present regarding the open nature of tackling skill and potential acquired knowledge of the experienced coach. At this point, this argument is logical, yet speculative, and it circumvents the issue at hand; in spite of the influence of the extensive familiarisation session, this small sample of raters did not show consistent reliability. It is thereby important to keep in mind the aim of developing a reliable tool for (wide-spread) use.

Thank you for your feedback and we appreciate your recognition of the complexities of assessing the reliability of observational tools. To confirm, an aim of the study was to conduct reliability analysis of the TackleTEK Tool within the specific context in which it was assessed, not for absolute generalisation of the results. We have tried to soften areas of the discussion where this might be misinterpreted (See lines 438-441. Your concern of the potential influence of coaching experience is understandable; however, in the context of this study, coaching experience was assessed directly. The number of raters used within this study was informed by previous reliability assessments of tackle competency tools in which reliability was tested using two or three raters (den Hollander et al., 2019; Waldron et al., 2014; Gabbett, 2008). We share your commitment to developing a reliable assessment tool for widespread application. We trust that many our previous responses also demonstrate that the approach to this paper is somewhat different to the reviewer’s conception of reliability.

L432: Strength of the conclusions based on the limited analytical goals…?

The conclusion was drawn from the implementation of multiple reliability measures of 1) the *a-priori* analytical goals, that were determined specifically for this study from pilot data to assess the reliability of the TackleTEK Tool within the specific parameters defined in this study, and 2) the non-parametric 95% limits of agreement test, that provides a comprehensive evaluation of rater agreement to offer a direct and interpretable measure of agreement, as an alternative to methods, such as the intraclass correlation coefficient, (see den Hollander et al., 2019; Gabbett, 2008), which is substantially more limited as reliability is determined by set thresholds rather than one set to determine its practical use within the tested context (Atkinson and Nevill, 1998).

L432-435: I cannot agree to the blanket statement that this tool can be used to assess tackling competency among schoolboys. This statement is factually inaccurate and somewhat misleading, perhaps evidencing (unconscious) investment bias or incentive-based bias. The tool demonstrated some promising features but the current design does not allow this tool to be labelled reliable in the overall context.

It is useful to avoid the term ‘fact’ in science, as it implies something is unquestionable, which is never the case. Thus, being factually accurate is not possible. We also do not appreciate the reviewer’s assumptions (at all) and ask that they alter their approach to reviewing the work of others. The reviewer should take a step back and try to appreciate the work of others’, as well as the statistical and methodological approach adopted here, which has clearly not been understood. We do understand the outside concern of the generalisability of the tool to assess tackling competency within schoolboy populations. The current process follows the recommended one, in that a measurement tool must be evaluated in context of their specific use, and generalisability should be considered in future research conducted with larger sample sizes (Atkinson & Nevill, 1998). We are happy to do this and are currently assessing much larger samples in this manner.

L436-437: I agree that more work is needed. The research presented is a good starting point but within the current research design certain structural limitation need to be addressed. A larger dataset within each rater tier-level would also provide for a stronger conclusion.

We agree this is a good starting point but, based on the reviewer’s comments, it appears that they do not fully understand why. We encourage them to read the papers we have cited throughout this rebuttal to enhance their own understanding.

Reference List

Atkinson, G., & Nevill, A. M. (1998). Statistical methods for assessing measurement error (reliability) in variables relevant to sports medicine. *Sports Medicine*, *26*, 217-238. DOI: [10.2165/00007256-199826040-00002](https://doi.org/10.2165/00007256-199826040-00002)

Cooper, S. M., Hughes, M., O’Donoghue, P., & Nevill, M. A. (2007). A simple statistical method for assessing the reliability of data entered into sport performance analysis systems. *International Journal of Performance Analysis in Sport*, *7*(1), 87-109. DOI: [10.1080/24748668.2007.11868390](http://dx.doi.org/10.1080/24748668.2007.11868390)

den Hollander, S., Lambert, M., Jones, B., & Hendricks, S. (2019). Tackle and ruck technique proficiency within academy and senior club rugby union. *Journal of Sports Sciences*, *37*(22), 2578-2587. DOI: [10.1080/02640414.2019.1648121](https://doi.org/10.1080/02640414.2019.1648121)

Gabbett, T. J. (2008). Influence of fatigue on tackling technique in rugby league players. *Journal of Strength & Conditioning Research, 22*(2), 625–632. DOI: <https://doi.org/10.1519/JSC.0b013e3181635a6a>

Haidet, K. K., Tate, J., Divirgilio‐Thomas, D., Kolanowski, A., & Happ, M. B. (2009). Methods to improve reliability of video‐recorded behavioral data*. Research in Nursing & Health, 32*(4), 465-474*.* DOI: [10.1002/nur.20334](https://doi.org/10.1002/nur.20334)

Hopkins, W. G. (2000). Measures of reliability in sports medicine and science. *Sports medicine*, *30*, 1-15. DOI: [10.2165/00007256-200030010-00001](https://doi.org/10.2165/00007256-200030010-00001)

McKay, A. K., Stellingwerff, T., Smith, E. S., Martin, D. T., Mujika, I., Goosey-Tolfrey, V. L., Sheppard, J., & Burke, L. M. (2021). Defining training and performance caliber: a participant classification framework. *International journal of sports physiology and performance*, *17*(2), 317-331. DOI: <https://doi.org/10.1123/ijspp.2021-0451>

Pollock, A. M., White, A. J., & Kirkwood, G. (2017). Evidence in support of the call to ban the tackle and harmful contact in school rugby: a response to World Rugby. *British journal of sports medicine*, *51*(15), 1113-1117. DOI: <https://doi.org/10.1136/bjsports-2016-096996>

Shill, I. J., West, S. W., Brown, J., Wilson, F., Palmer, D., Pike, I., Hendricks, S., Stokes, K. A., Hagel, B. E., & Emery, C. A. (2023). How to harness and improve on video analysis for youth rugby player safety: a narrative review. BMJ open sport & exercise medicine, 9(3), e001645. DOI: [10.1136/bmjsem-2023-001645](https://bmjopensem.bmj.com/content/bmjosem/9/3/e001645.full.pdf?utm_source=chatgpt.com)

Smith, S. E., McColgan-Smith, S., Stewart, F., Mardon, J., & Tallentire, V. R. (2024). Beyond reliability: assessing rater competence when using a behavioural marker system. *Advances in Simulation*, *9*(1), 55. DOI: <https://doi.org/10.1186/s41077-024-00329-9>

Spitz, J., Moors, P., Wagemans, J., & Helsen, W. F. (2018). The impact of video speed on the decision-making process of sports officials. *Cognitive Research: Principles and Implications*, *3*, 1-10. DOI: [10.1186/s41235-018-0105-8](https://cognitiveresearchjournal.springeropen.com/articles/10.1186/s41235-018-0105-8)

Waldron, M., Worsfold, P. R., Twist, C., & Lamb, K. (2014). The relationship between physical abilities, ball-carrying and tackling among elite youth rugby league players. *Journal of sports sciences*, *32*(6), 542-549. DOI: [10.1080/02640414.2013.841975](http://dx.doi.org/10.1080/02640414.2013.841975)

Wilkie, B., Jordan, A., Foulkes, J., Woods, C. T., Davids, K., & Rudd, J. (2023). Examining the validity, reliability and feasibility of capturing children's physical literacy through games-based assessment in physical education. *Frontiers in Sports and Active Living*, *5*, 1188364. DOI: <https://doi.org/10.3389/fspor.2023.1188364>
